# Experiences With Technology Among Adults Aging With HIV Engaged in an Online Community–Based Exercise Intervention Study: Longitudinal Qualitative Descriptive Study and Secondary Data Analysis

**DOI:** 10.2196/86785

**Published:** 2026-07-02

**Authors:** Julia Mucha, Rana Hamdy, Melina Marini, Reda Aasem, Chung Duong, Tai-Te Su, Soo Chan Carusone, Kelly K O'Brien

**Affiliations:** 1 Department of Physical Therapy, Temerty Faculty of Medicine, University of Toronto, 160-500 University Avenue, Toronto, ON, M5G 1V7, Canada, 1 416-978-0565; 2 School and Graduate Institute of Physical Therapy, College of Medicine, National Taiwan University, Taipei, Taiwan; 3 McMaster Collaborative for Health and Aging, McMaster University, Hamilton, ON, Canada; 4 Institute of Health Policy, Management and Evaluation (IHPME), Dalla Lana School of Public Health, University of Toronto, Toronto, ON, Canada; 5 Rehabilitation Sciences Institute (RSI), University of Toronto, Toronto, ON, Canada

**Keywords:** HIV, exercise, telerehabilitation, telehealth, digital health, wearable technology, online coaching

## Abstract

**Background:**

As individuals with HIV live longer, many now face the health consequences of aging and multimorbidity, known as disability. Exercise can mitigate disability; however, engagement in exercise among adults living with HIV varies. Technology-based interventions, such as telerehabilitation, may help mitigate geographical, financial, and time barriers to community-based exercise (CBE). However, little is known about the experiences with technology uptake and usage among adults living with HIV. Understanding these experiences is essential to inform the design of inclusive, accessible, and sustainable online interventions.

**Objective:**

This study aimed to describe experiences with technology uptake and usage among adults aging with HIV participating in a 6-month online CBE intervention and explore how these experiences changed over time, from baseline to postintervention.

**Methods:**

We conducted a longitudinal qualitative descriptive study and secondary analysis using interview data from adults living with HIV who were engaged in a CBE intervention study in Toronto, Canada. Participants engaged in a 6-month online CBE intervention consisting of thrice-weekly exercise supervised biweekly through online personal coaching sessions, weekly group exercise classes, and monthly self-management education sessions (via Zoom). The technology used included Zoom software and a webcam, as well as the Sweat for Good YMCA app and the YMCA Virtuagym website; participants wore a wireless physical activity monitor (Fitbit Inspire 2) throughout. Participants completed interviews at baseline and postintervention. We conducted a group-based content analysis of interview transcripts, focusing on digital access, setup, usage, and perceptions of technology. Questionnaire data describing digital literacy and access to technology provided additional context to the interview data.

**Results:**

Eleven participants completed at least one interview. We analyzed 19 interview transcripts from 11 participants (women: n=6, 55%; men: n=5, 45%; median age 52, IQR 45-60 y). Experiences with technology uptake and usage among adults aging with HIV were characterized by four components: (1) preparations for technology (technology setup), (2) interactions with technology (preferences for different types of technology, preferences for mode of delivery, and ease of usage), (3) facilitators and satisfaction with technology (facilitators to technology uptake and usage and satisfaction with technology), and (4) challenges and frustrations with technology (barriers to technology uptake and usage and frustrations with technology). Experiences with technology across participants were influenced by intrinsic contextual factors (prior exposure to technology) and extrinsic contextual factors (COVID-19 pandemic and technological and social support).

**Conclusions:**

Experiences with technology among adults aging with HIV engaging in an online CBE intervention varied from increasing ease of use to increasingly burdensome over time. Results highlight the need to incorporate personal preferences and ongoing technological support when implementing online CBE with adults aging with HIV.

## Introduction

Approximately 65,000 people in Canada are living with HIV as of 2022, with an estimated 40.8 million people globally living with HIV at the end of 2024 [1,2]. Antiretroviral therapy has enabled people with HIV to live longer; however, many may also be living with associated multimorbidity affecting cardiovascular, autoimmune, musculoskeletal, and neurodegenerative systems [3,4]. These health challenges constitute disability that may manifest as episodic over time [5].

The Episodic Disability Framework conceptualizes disability as multidimensional and episodic, characterized by periods of wellness and illness [5,6]. The framework includes 6 dimensions of disability experienced by adults aging with HIV: physical, cognitive, and mental-emotional symptoms and impairments; difficulties with day-to-day activities; challenges to social inclusion; and uncertainty or worry about the future [5,6]. Intrinsic contextual factors (eg, personal attributes and coping strategies) and extrinsic contextual factors (eg, social support and stigma) can exacerbate or alleviate disability over time [5]. This framework provides a foundation to understand the health challenges experienced by adults living with HIV and the strategies to mitigate episodic disability.

Exercise and physical activity are rehabilitation strategies that can mitigate episodic disability among adults aging with HIV. Physical activity is defined as any type of bodily movement that results in energy expenditure, while exercise is a subset of physical activity that is planned, structured, and repetitive, with a goal of improving or maintaining physical fitness [7]. Systematic reviews indicate that adults living with HIV who engage in exercise at least three times weekly can experience improvements in cardiopulmonary fitness, strength, and quality of life [8-11]. The World Health Organization (WHO) recommends that adults, including persons living with chronic conditions, such as HIV, should engage in at least 150 minutes of moderate to vigorous intensity aerobic physical activity per week alongside muscle-strengthening activities on 2 or more days [12]. Despite the benefits, engagement in exercise can vary among adults living with HIV.

Systematic review evidence indicates that only 51% of adults living with HIV met exercise guidelines of 150 minutes of moderate to vigorous aerobic exercise weekly [13,14]. Barriers to exercise engagement were multifactorial, encompassing personal factors (lack of motivation, time constraints, and health concerns), social barriers (competing priorities and HIV-related stigma), and environmental challenges (transportation and financial constraints) [15-18]. Community-based exercise (CBE) programs can facilitate engagement in exercise among adults living with HIV, fostering social interaction and self-management strategies through organized exercise programs under supervision in community-based facilities [19,20]. In-person CBE can improve cardiovascular health, strength, and flexibility outcomes among adults living with HIV [21]. However, the complex and episodic nature of HIV, coupled with stigma, body image concerns, geographical barriers, and scheduling conflicts, can limit the engagement of persons living with HIV with in-person CBE programs [17].

Telerehabilitation, the delivery of rehabilitation services remotely using technologies, offers promising solutions to geographical, financial, and time barriers limiting access to exercise interventions [22-26]. Telecoaching, a component of telerehabilitation, uses technology for remote supervision and guidance to deliver an exercise program to improve physical activity levels and symptom management in chronic diseases [27-29]. Technology is an integral part of telecoaching CBE interventions to facilitate engagement in exercise; however, experiences with the uptake and usage of technology among adults aging with HIV engaged in such an intervention are unknown.

Technology encompasses digital tools designed to streamline care delivery, increase exercise engagement, and improve health outcomes [30]. This includes telecommunication platforms, web applications, and wireless physical activity monitors (WPAMs). Technology uptake refers to the initial adoption of technology by users, whereas usage refers to how individuals apply these tools, considering the frequency, functionality, effectiveness, and adherence of use [31]. Among adults aging with HIV, technology can support health-promoting behaviors and exercise engagement [25,26,32]. Systematic reviews highlight the role of technology in enhancing group exercise participation through social support and can help to alleviate barriers by addressing geographical, financial, and time constraints [33,34]. Technology-based exercise interventions are supported for use in chronic conditions such as Parkinson disease, chronic obstructive pulmonary disease, and stroke [27,29,35]. Despite positive trends in technology-facilitated exercise interventions, it is unclear how technology is taken up and used among adults aging with HIV.

Several factors can influence technology uptake and usage among adults living with chronic conditions, including prior exposure to, and awareness of, types of technologies, digital literacy, accessibility, and required support for use [36-39]. In HIV-specific contexts, there is growing interest in using technology to support engagement in exercise [32,40-42]. The purpose of this study was to describe experiences with technology among adults aging with HIV engaged in an online CBE intervention. The specific objectives were to describe (1) experiences with technology uptake and usage and (2) how experiences with technology may change over time among adults aging with HIV engaging in an online 6-month CBE intervention.

## Methods

### Study Design

We conducted a longitudinal qualitative descriptive study and secondary analysis using interviews and questionnaire data from adults aging with HIV who participated in a 6-month online CBE intervention (Tele-Coaching CBE Study) [42]. Our qualitative design was supported by quantitative (questionnaire) data collected at baseline, month 2 (after initial exposure to the intervention), and month 6 (post intervention).

### Context: Telecoaching CBE Study

The telecoaching CBE study was a prospective longitudinal mixed-method intervention study designed to evaluate the implementation of a 6-month online telecoaching CBE program for adults aging with HIV, delivered in collaboration with the Toronto YMCA [42]. Participants were community-dwelling adults living with HIV who self-identified as safe to engage in exercise based on the Physical Activity Readiness Questionnaire. Recruitment occurred through the Ontario HIV Treatment Network Cohort Study and community-based organizations in Toronto [42,43].

In phase 1 (intervention phase), participants were expected to engage in 60 minutes of exercise thrice weekly. Sessions were supervised biweekly by a YMCA personal trainer via Zoom, and participants were asked to attend monthly online self-management sessions for 6 months. In phase 2 (independent phase), participants continued with unsupervised exercise thrice weekly for 6 months. Participants were provided with a WPAM (Fitbit Inspire 2; Fitbit Inc) to track steps, distance, calories burned, and active minutes. Throughout both phases, participants received a YMCA online membership that provided access to synchronous group exercise classes via Zoom and recorded workouts (also called Avatar Workouts), YMCA Virtuagym website, and the Sweat for Good YMCA app. An engagement coordinator assisted participants with the setup of the technology and was available to provide support as needed throughout the intervention.

Investigators of the online CBE study conducted qualitative interviews via Zoom with a subset of participants at baseline (time point 1 [T1]) and postintervention (time point 2 [T2]) to explore their experiences and perceived impact of the intervention [44]. Full protocol details for the online CBE study have been published [42]. In our study, we focus on interview data pertaining to participants’ experiences with technology during phase 1 of the telecoaching CBE study.

### Technology in Online CBE Intervention

Technology was integral to the online CBE study, serving roles in (1) intervention delivery, (2) facilitating engagement in physical activity, and (3) measuring outcomes. For the purpose of this study, we defined technology as the *tools* (hardware, software, networks, and web applications) and *processes* (methods and strategies used for instruction, assessment, and physical activity tracking) used in the telecoaching CBE study. Key technological components included Zoom software (Zoom Communications, Inc) and a webcam for online personal training sessions and self-management education sessions; Sweat for Good YMCA app and YMCA Virtuagym website for live group exercise classes, prerecorded workouts, and recorded personalized workouts created by a personal trainer; WPAM (Fitbit Inspire 2) for motivation and measurement of physical activity; and technology-supported assessments, including online fitness assessments and interviews via Zoom, administration of patient-reported outcome measures via Qualtrics web-based survey software, body composition scale, and Fitbit app for syncing activity data [42]. In this study, we focused on experiences with technology pertaining to components of the intervention delivery.

### Participants and Recruitment

Eligible participants for the telecoaching CBE study were required to have access to (1) a cell phone, tablet, laptop, or desktop computer; (2) internet access via Wi-Fi or a wired connection; (3) a webcam; and (4) a personal space for exercise. A subset of the participants in the telecoaching CBE study was invited to participate in the qualitative component of the study. Women living with HIV were purposively recruited to the qualitative study to enhance gender diversity within the sample.

In our study, we included community-dwelling adults aging with HIV (≥18 y) in Toronto, Canada, who considered themselves medically stable and who were safe to participate in the telecoaching CBE study and completed at least one qualitative interview at either baseline (T1) or postintervention (T2).

### Patient and Public Involvement

We consulted with 3 community experts who self-identified as living with HIV. These consultations provided contextual insights into exercise and technology experiences within both in-person CBE and online CBE interventions. We met with each community expert via Zoom to discuss their experiences related to exercise engagement, intervention process, technology usability, and feasibility. These consultations provided foundational information for understanding the intervention and technology use and ensured our analysis and interpretation of interview data were informed by persons living with HIV.

### Data Sources

#### Interviews

We used interview transcripts from semistructured interviews conducted at baseline (T1) and postintervention (T2) within the online CBE study to explore participants’ experiences with technology uptake and usage and how experiences changed over time. The interviews were completed from October 8, 2021, to July 28, 2022, by members of the research team. We reviewed full transcripts, focusing our analysis on data that pertained to technology experiences. Interview questions at baseline about technology uptake and usage included: “Do you have any concerns or perceived challenges of participating in the upcoming CBE intervention?” and “How do you feel about adopting the technology necessary for the upcoming online CBE telecoaching intervention?” Interview questions at T2 focused on technology experiences, such as: “How would you describe your experience using the technology as part of the CBE telecoaching intervention?” and “Have there been any strengths or challenges associated with the translation of the telecoaching CBE intervention into the HIV community?” Interviewers used open-ended general and probing questions to explore technology uptake, learnability, usability, and change in experiences over time. Relevant interview guide questions are highlighted in Supplemental File 1 in Multimedia Appendix 1. Participants received a $30 CAD (US $23) electronic gift card for their participation in each interview.

#### Questionnaires

We used 3 questionnaires to capture participant demographics and describe technology access characteristics, digital literacy, and telehealth experiences. The demographic questionnaire (administered at baseline) was used to describe participants’ personal, health, and exercise behavior characteristics. The Information and Communications Technologies Development Index Questionnaire (administered at baseline) included 11 items to describe participants’ digital access, connectivity, and literacy [45,46]. The Telehealth Indicator Questionnaire (administered at months 2 and 6) included 22 items with the aim to assess technologies used as part of the online CBE intervention across key telehealth domains, including usability (ease of use and navigation; 7 items), helpfulness (6 items), satisfaction (6 items), and reliability (3 items) [45,47].

### Data Analysis

#### Interview Data

We conducted a collaborative group–based qualitative content analysis using a 6-step approach informed by the DEPICT model [48]. Five team members (JM, RH, MM, CD, and RA) independently read all transcripts, followed by group discussions to identify emerging codes related to technology uptake and usage. A preliminary codebook was developed based on the study objectives and operationalized technology experiences from the online CBE intervention study. We piloted the codebook on 3 transcripts with the most technology-related content. The team met 6 times to refine code definitions, incorporate barriers and facilitators to technology uptake and usage, and eliminate redundancies of codes. After 3 iterative revisions, 2 team members independently coded each transcript [48]. We used NVivo 15 software for data management [49]. For each participant, we wrote brief summaries of their technology experiences and interpretations of change over the intervention with the aim to identify patterns in how participants interpreted technology use and its impact over time. Our analytical process included team discussions for clarification, critical reflection, and consensus building [48]. To identify technology experiences over time, we coded each T1 and T2 transcript concurrently. We summarized individual experiences, documented reflections on changes in experiences, and identified data reflecting temporal changes. We discussed interview findings with questionnaire data to provide contextual interpretations.

#### Questionnaire Data

We analyzed the questionnaire data using Microsoft Excel [50]. For the Information and Communications Technologies Development Index Questionnaire, Telehealth Indicator Questionnaire, and demographic questionnaire, we calculated frequencies and percentages for categorical variables, and medians with 25th and 75th percentiles for continuous variables. For the Telehealth Indicator Questionnaire, we assessed the direction of change in responses between months 2 and 6 when responses differed between time points. For example, in the “satisfaction” domain, a change from “excellent” to “poor” indicated a negative change in participants’ experiences with technology. Supplemental File 2 in Multimedia Appendix 2 provides the Telehealth Indicator Questionnaire. We included all questionnaire data where available at each time point.

We analyzed the interview and questionnaire data concurrently using descriptive approaches. We used the questionnaire data to inform our content analysis of the interview data, specifically by providing additional context to participant personal and health characteristics and their access to and familiarity with technology to enhance our interpretations and deepen our understanding of experiences with uptake and usage of the technology.

### Sample Size Justification

We had a fixed sample of 11 participants from the online CBE study who completed at least one interview at T1 or T2. Of these, 8 participants completed both T1 and T2 interviews, 2 participants completed the T1 interview only, and 1 participant completed the T2 interview only. This sample size is comparable to those of prior qualitative studies examining technology experiences in telehealth exercise interventions among individuals with chronic conditions [51,52].

### Ethical Considerations

Ethics approval for this study (protocol 47552) and the broader telecoaching CBE study (protocol 40410) was obtained from the University of Toronto Health Sciences Research Ethics Board. All participants in the telecoaching CBE study who completed an interview provided written or verbal informed consent. Data sources including transcripts and questionnaires were deidentified, meaning participant data were identified using a participant number to ensure confidentiality of participants. Participants received a CAD $30 (US $23) electronic gift card for their participation in each interview.

## Results

### Characteristics of Participants

Of the 11 participants (median age 52, IQR 45-60 y), 6 (55%) identified as women, 5 (45%) identified as heterosexual, and 6 (55%) identified as Black. The majority of participants (n=9, 82%) had an undetectable viral load. Five (45%) participants reported living with ≥3 concurrent health conditions in addition to HIV. Exercise behavior among participants prior to the intervention varied. Three (27%) participants did not engage in exercise in the past week, 5 (45%) engaged in 1 to 2 days, and 3 (27%) engaged in 5 or 7 days of ≥30 minutes of moderate to vigorous intensity exercise in the past week. Table 1 provides participant characteristics.

**Table 1.** Characteristics of participants (n=11).

Characteristic	Participants (n=11)
Personal characteristics	
Age (y), median (25th, 75th percentile)	52 (45, 60)
Sex, n (%)	
Female	6 (55)
Male	5 (45)
Gender, n (%)	
Woman, cis-woman	6 (55)
Man, cis-man	5 (45)
Sexual orientation, n (%)	
Heterosexual	5 (45)
Gay	3 (27)
Queer	1 (9)
Bisexual	1 (9)
Other	1 (9)
Living arrangement, n (%)	
Living with others	7 (64)
Living alone	4 (36)
Average gross yearly income (CAD), n (%)	
<$20,000	4 (36)
$20,000 to <$50,000	5 (45)
>$50,000	2 (18)
Primary source of income, n (%)	
Ontario Disability Support Program	4 (36)
Full-time employment	3 (27)
Pension plan	2 (18)
Part-time employment	1 (9)
Casual work	1 (9)
Education level, n (%)	
Completed college (received degree or diploma)	5 (45)
Completed university (received degree)	3 (27)
Postgraduate education	3 (27)
Ethnicity^a^, n (%)	
Black	6 (55)
South Asian	3 (27)
Latin American, Hispanic, or Latino	1 (9)
Southeast Asian	1 (9)
White	1 (9)
Clinical characteristics	
Year of HIV diagnosis, median (25th, 75th percentile)	2002 (1995, 2006)
Taking antiretroviral medication, n (%)	11 (100)
Number of missed doses in past 7 d, n (%)	
No doses missed	7 (64)
1 dose missed	4 (36)
Undetectable viral load (<50 copies/mL)	9 (82)
Recent CD4 count, n (%)	
>500 cells/mm^3^	9 (82)
<500 cells/mm^3^	2 (18)
Number of participants with ≥3 comorbidities, n (%)	5 (45)
Self-reported health status, n (%)	
Very good	7 (63)
Good	2 (18)
Fair	1 (9)
Poor	1 (9)
Exercise behavior characteristics, n (%)	
Days per week physically active ≥30 min at a moderate or vigorous intensity in the past week prior to intervention, n (%)	
0 (none)	3 (27)
1 d	2 (18)
2 d	3 (27)
5 d	1 (9)
7 d	2 (18)
Engaged in >300 min of moderate intensity activity or >150 of vigorous intensity activity in the past week prior to intervention, n (%)	2 (18)
Impact of COVID-19 pandemic on physical activity, n (%)	
Exercises at a greater frequency or intensity	5 (45)
Exercises at a lesser frequency or intensity	5 (45)
No impact on exercise	1 (9)

a One participant listed 2 ethnicities.

### Access To, Skills in, and Use of Technology at Baseline

At baseline, most participants had access to a cell phone (n=10, 91%), a laptop with a webcam (n=9, 82%), and reliable Wi-Fi access (n=9, 82%). All participants (100%) reported they were comfortable using web browsers, online meeting tools, WPAMs, and fitness apps at baseline. Table 2 provides participant characteristics regarding access to, skills in, and use of technology.

**Table 2.** Access to, skills in, and use of technology at baseline (N=11).

Information and communications technologies questionnaire variable	Participants, n (%)
Access to technology	
Access to a type of technology	
Cell phone	10 (91)
Laptop computer with webcam	9 (82)
Tablet	5 (45)
Desktop computer with webcam	4 (36)
Desktop computer without webcam	0 (0)
Most reliable access to the internet	
Through Wi-Fi	9 (82)
Through a wired connection	2 (18)
Skills and use of technology	
Comfortable using the World Wide Web with a browser	11 (100)
Comfortable sending and receiving emails	11 (100)
Comfortable using online meeting tools	11 (100)
Comfortable using wireless physical activity monitor	11 (100)
Comfortable using webcam	11 (100)
Comfortable using physical activity apps to track physical activity	11 (100)
Confirmed skills and use of technology	11 (100)

### Experiences With Technology Uptake and Usage

**Figure 1.**
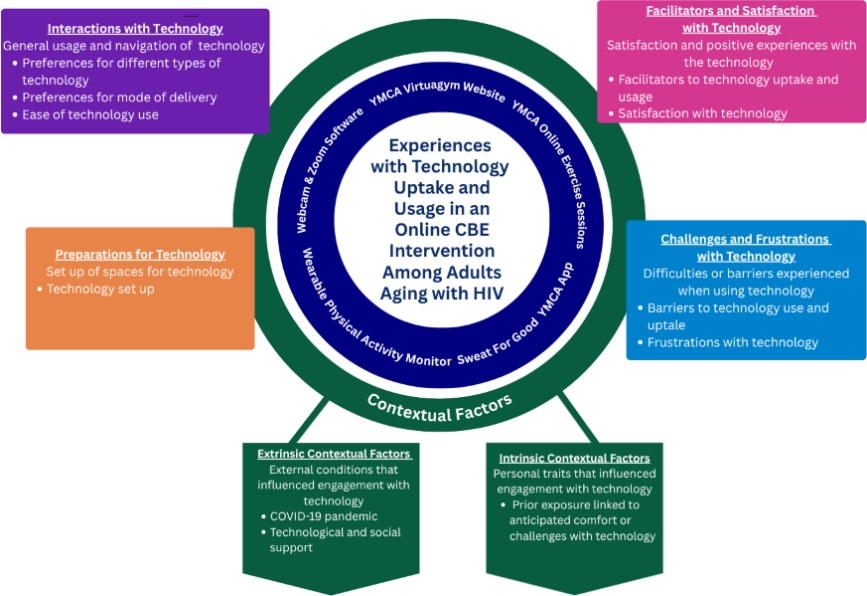
Experiences with technology uptake and usage in an online community-based exercise (CBE) intervention among adults aging with HIV.

Participants’ experiences with technology during the online CBE intervention varied. We organized our findings into four interrelated components: (1) preparations for technology, (2) interactions with technology, (3) facilitators and satisfaction with technology, and (4) challenges and frustrations with technology. Participants’ experiences with technology were shaped by both intrinsic contextual factors, such as their prior exposure and anticipated comfort with technology, and extrinsic contextual factors, including the COVID-19 pandemic and availability of technological and social support. These factors affected how participants engaged with the technology (uptake and usage) in the study, as well as the benefits and challenges they experienced (Figure 1). Below, we present each component with supportive quotations identified by participant number and interview time point.

#### Preparations for Technology

This component captures how participants prepared their physical spaces and devices to engage with the study. Preparation was not limited to technical setup; it also involved adapting the home environments to accommodate space for exercise and facilitate active participation. These preparatory steps often required creativity and problem-solving. Most participants reported having adequate space for exercise, although some required modifications, as illustrated below:

*Other than the sense to figure out where my laptop would be so that the instructor could see me and figure out how my apartment would work. It’s a small apartment... I wish I didn’t have to use my box [exercise equipment] because it would be great to put my laptop on to get the whole view of my workout area. But no. I think I’ll figure it out. I’ve just got to move some things, just change the angle of some things.*

[P2, T1]

The following participant described their experiences trying to set up their Zoom to the TV for optimal viewing of their personal trainer during the online session:

*I’m hoping I can run a Zoom meeting off my iPhone. But I think what I’m going to need is get a cable to connect to the HDMI cable on the TV. So it’s just a bit of technical stuff to get all that set up. I’ve got a little tripod that I can set the phone on. The microphone will work through the phone. It will just be a question of running the display out of the phone with a cable into the HDMI connection.*

[P5, T1]

#### Interactions With Technology

Participants described a variety of experiences interacting with technology, reflecting differences in preferences, confidence, and adaptability. Participants’ descriptions of their experiences interacting with technology focused primarily on preferences for different types of technology, preferences for mode of delivery, and ease of technology use.

The preferences for types of technology participants used included Zoom, Fitbit, computer, laptop, webcam, Fitbit app, and Sweat for Good YMCA app. Zoom was the primary platform for online telecoaching sessions with the personal trainer, with most participants accessing Zoom via a laptop or desktop with a webcam, while a smaller number preferred mobile phones or connecting Zoom to their TVs to enhance convenience or visibility, respectively.

The Fitbit device and the Fitbit app were largely used to track step counts by most participants. The following participant explained the use of the Fitbit:

*I like wearing it [Fitbit] but not for the reason for exercise because I don’t understand anything on it. I like it for two reasons; number one, I can see steps and number two I can see time [...]. Besides that, because I don’t know technology, I didn’t really benefit from the usefulness of having a Fitbit that it can tell you more stuff. But for these two reasons it was good enough for me*.

[P11, T2]

The Sweat for Good YMCA app was used to attend online group exercise classes, prerecorded workouts, and the Avatar workouts, offering participants flexibility with both synchronous and asynchronous options. Preferences for mode of delivery varied between synchronous and asynchronous modes of online exercise delivery. Some found the synchronous Zoom training sessions straightforward, while others found it stressful due to technical challenges and preferred the convenience of the asynchronous classes of the Sweat for Good YMCA app. The following participant struggled with navigating the Sweat for Good YMCA app stating:

*However, first of all trying to get everything set up for a synchronous event I just found it incredibly stressful again because of trying to navigate the technical challenges. Then when I saw the instructor on the screen they were doing some warmup things at a pace that I could barely keep up with*.

[P5, T2]

This same participant (P5) specifically struggled to maintain pace during the synchronous exercise class through the Sweat for Good YMCA app, stating:

*So the synchronous thing, it just wasn’t for me. Really the thing that has most worked for me is just knowing routines that [name of personal trainer] has given me and writing them down and keeping up with that on my own. The less I have to interface with technology in this kind of remote setup, the better*.

[P5, T2]

Ease of technology use referred to how easy participants found the technology to be. Many participants anticipated positively adopting the new technology at T1. However, their actual ease of use varied once they began engaging with the technology. This participant describes their experiences with their ease-of-use learning about the technology:

*With me in trying to figure out technology, it was straightforward. Like once I plugged in to charge it, it told me to download the App and then once I did that, it was a step by step. It’s more still figuring out the features. Like, I had no idea that it already recorded my sleep overnight until my roommate and bestie looked it up and was like okay*.

[P2, T1]

Another participant was surprised to find the use of the technology to be challenging and required additional support to navigate:

*Well initially… and this kind of took me by surprise. I didn’t realize how difficult the technical part of things would be. It was horrendously difficult, the technical part. In fact, AM, we gave up an entire hour of training because AM got her daughter involved on her daughter’s laptop, and we went through setting by setting to try to eliminate the terrible echo. You know I’m a big advocate of clear communication and Zoom has done a terrible job and most software providers do a terrible job of explaining things.*

[P5, T2]

#### Facilitators and Satisfaction With Technology

This component explores what helped participants successfully adopt and use technology and how these factors contributed to their satisfaction. Facilitators ranged from supported features such as activity tracking and sharing their progress in social circles to practical benefits such as convenience and cost savings. These elements often reinforced participants’ motivation and sense of accountability.

One participant described using the Fitbit to stay accountable:

*So my mom has a Fitbit too and I added her as a family member or friend. So now she gets to see how much I am doing for the day. There will be days that she’ll talk to me or whatever or like I’ll send her my badges that I’ve won*

[P2,T2]

The following participant appreciated not having to commute to a gym facility. They highlighted the convenience and cost-saving benefits of using Zoom rather than having an in-person personal training session:

*The advantage of just having to get up and not have to drive anywhere. Just get up and you know have your Zoom on. So that was very convenient, very convenient. Yeah. I’m not spending gas. Like gas prices are ridiculous right now*.

[P1, T2]

Satisfaction with technology referred to participants’ approval with the online platforms they engaged with through the CBE intervention. Some participants indicated that they were satisfied with having access to the tools that supported their goals. Several participants described the Fitbit as helpful for monitoring their progress with physical activity. Many participants found that the weekly summary reports from Fitbit were rewarding and motivated their continued engagement:

*A part of the study that worked best for me. For me I would say like I have best... okay definitely Fitbit. Fitbit is like... not only having the Fitbit. It helped me to track all my things right; the steps, those all sleeping patterns and even like weekly we have to something that report. Those are all in the Fitbit Apps. So those things that, seeing those all tracked, So those things that, seeing those all tracked, it satisfied me. It motivates me. I want to see like in the number or in the papers and these apps really help give me all those things what I’m looking*

[P8, T2]

One participant talked about how features of the Fitbit as well as the online platforms encouraged their participation:

*It’s sort of like an encouragement to do more. These watches are good because I’ve sort of got used now to whenever I’m out walking to enter the time. The classes, the sessions or lectures that you have are very good.*

[P3, T2]

#### Challenges and Frustrations With Technology

Participants also experienced challenges and frustrations with the study technology. At times, these challenges hindered engagement in components of the intervention.

Zoom was generally perceived as a useful platform; however, some participants reported technical challenges, including audio delays, echoing, and unstable internet connections. At time point 1, one participant shared their concerns about the stability of their internet:

*In the neighborhood I live in, the internet has gone down I don’t know maybe two handfuls of a time within the past six months. So you never really know.*

[P2, T1]

Another participant talked about the lack of accessibility of the Fitbit features:

*The second is if you’ve got any issues around sight, any, I mean even if you don’t have issues around sight, you can’t see these things. You go into the... you go outside, it’s... so like you do your walk and you’re finished and you come back and you go am I at the screen where it’s turned off? You can’t tell because you can’t read it. And of course the writing is so small that people that have impaired vision, even if you can see it, it’s difficult to read.*

[P10, T2]

Frustrations with technology were tied to the reliability of the technology and to design limitations. This included Zoom audio delays, sound issues, echoing, and connectivity problems. Participants noted that Zoom provided insufficient support, requiring them to troubleshoot issues independently, as per the following participant:

*It’s just technical stuff, those little quarks about my particular setup. There was a time when I was having audio issues, and it was downright stressful to be there [self-management sessions] for the hour and a half-*

[P5, T2]

Another participant shared their frustrations related to the Fitbit, including device setup, blank display screen, and concerns about inaccurate tracking:

*The biggest demotivator for this whole thing is that stupid Fitbit that they gave us, utterly useless, good for nothing, utterly useless. The only thing I use it for is to count whether it’s counting the number of steps. That’s it. Every month since I got it there’s been some issue with it. For some reason it went into some other country time on its own and then at some point it just completely the screen was blank and it dimmed on its own at some point. Every time it does something on its own.*

[P7, T2]

#### Contextual Factors

Participants’ experiences with technology were shaped by a range of contextual factors that influenced both uptake and ongoing use of technology in the online CBE intervention. Together, intrinsic and extrinsic contextual factors influenced how participants approached technology, the challenges they anticipated, and the strategies they used to overcome barriers.

Intrinsic contextual factors included prior exposure to technology that influenced a participant’s anticipated comfort or challenges with the forthcoming technology in the online CBE intervention. Six (54.5%) of the 11 participants previously or currently owned WPAMs, such as an Apple Watch, Samsung Watch, or a Fitbit. Many participants had previously used Zoom prior to the intervention. Participants with prior exposure to technology often reported increased confidence of use at baseline and anticipated greater comfort with technology use, as this participant stated:

*That’s okay. I have a degree in computer sciences so it’s not an issue.*

[P7, T1]

When another participant was asked about perceived challenges regarding updating software with technology associated with the CBE intervention, they responded:

*No. As far as updating the equipment and stuff I’m fairly... that goes fairly easy for me.*

[P10, T1]

Extrinsic contextual factors included the COVID-19 pandemic and available technical and social support throughout the intervention. Many participants reported that the COVID-19 pandemic normalized the use of technologies, such as Zoom. For some participants, the COVID-19 pandemic led them to their first exposure to technology as it forced them to engage with technology in new ways. This participant reflected on both the safety and convenience of technology as well as the convenience of exercising from the comfort of their own home:

*One is the times that we’re in with COVID, I mean I think there’s a safety component to this. …. for a lot of people too because a person’s immunity is a vital part of the constitution. I think the convenience of being able to just be at home and in the comfort of your own home is helpful.*

[P10, T1]

Another participant acknowledged the motivational pull that comes from an in-person gym setting yet still preferring online sessions for safety concerns that began during the COVID-19 pandemic:

*To be honest while in the pandemic I prefer to going online. That way I’m safe, I’m good. But also going into the gym and seeing others working out kind of pushes you like listen that person can do it, why can’t I. So, it kind of gives you that push compared to online because online is just me and my trainer. I know they will probably push you but it’s not the same as actually seeing somebody else, maybe somebody else bigger or somebody older actually doing it easier than you. So, it kind of gives you that push. But I’m more than happy with online right now.*

[P7, T1]

Availability of technical and social support played a key role in how some participants planned to address technical challenges in the study. Some participants mentioned the technology support services having insufficient troubleshooting resources and resulting in alternative methods to troubleshoot. This participant reported:

*I wouldn’t say they [technical issues] become an obstacle. Normally when I can’t figure out something I just Google or go on YouTube and figure out what it is, what’s going on and how to fix it.*

[P6, T1]

Other participants depended on their social support network to assist with device setup or to resolve technical issues. When asked about navigating technical difficulties at home, 1 participant shared how they relied on their children for assistance:

*If I can’t function something I just call them, ‘hey show me how this works.*

[P1, T1]

### Changes in Experiences With Technology Within the CBE Intervention Over Time

Interview data revealed participants had inconsistent experiences with uptake and usage of technology over the duration of the intervention. Some participants experienced increasing levels of technology fatigue and burden over the course of the intervention. Some participants found technology a challenge at both T1 and T2. For 1 participant, by T2, their concerns evolved into frustrations with the technology expressing technology fatigue and burden, particularly with the requirement to download and sync their physical activity each week for the study:

*This synching up for the Fitbit and the recording on what’s gone on over the week, it gets to be a bit of a pain to be honest.*

[P5, T2]

Although participants attempted to problem-solve their challenges, frustrations continued with efforts to use the Fitbit. The requirement to sync their Fitbit data weekly became a burden and at times resulted in disengagement and frustration. In contrast, another participant was optimistic with using the Fitbit at T1:

*Having the coach itself is a very good benefit right...it will motivate me -- oh I’m wearing a Fitbit, so I have to do, let’s just do it’ and also do the exercise.*

[P8, T1]

By T2, this participant’s expectations appeared to have been met, and they expressed their intent to continue using the technology beyond the study*:*

*even giving this Fitbit, I will use it unless it will not damage. If it damage, then definitely I’m going to buy another one because Fitbit gives all that report.*

[P8, T2]

Another participant expressed uncertainty about technology usage in T1 and had an indifferent attitude toward the Fitbit and its role in the intervention, stating:

*I mean I think they are all the same. They don’t have any difference. So I don’t know. Like what information do you want from these watches?*

[P3, T1]

However, by T2 their perspectives regarding the Fitbit had changed:

*It’s good. Fitbit is… after the first or second time that you enter the information online it becomes very easy. Yeah, it’s good.*

[P3, T2]

This participant experienced a learning curve but was able to reduce barriers and increase usability of the Fitbit with continued use. Overall, initial attitudes toward the technology did not always predict participants’ engagement with participants’ engagement or satisfaction during the study. Some participants encountered technology fatigue around repetitive tasks such as Fitbit syncing, while others reported increased comfort and motivation.

#### Telehealth Indicator Questionnaire Responses

Eight (73%) participants completed the Telehealth Indicator Questionnaire at months 2 and 6 of the intervention. Across the 8 participants, the majority (n=6, 75%) demonstrated no change in most responses to the 22 items at months 2 and 6. Two (25%) participants had a change in a positive direction at month 6 in 10 (46%) and 12 (55%) of the 22 items. Supplemental File 3 in Multimedia Appendix 3 provides each participant’s direction of responses to the 22 items of the Telehealth Indicator Questionnaire, related to usability, helpfulness, satisfaction, and reliability of the technology in the CBE intervention captured at months 2 and 6.

Across the 22 items, the majority of participants (50%‐88%) reported similar responses (representing no change) for most items (14/22, 63%) at months 2 and 6 of the intervention. An exception was responses to the usability and helpfulness of self-directed learning modules, whereby 5 participants (62%) demonstrated a positive direction of change in their response from months 2 to 6. Conversely, 4 (50%) participants showed a negative change in direction in responses to the usability of the Fitbit and helpfulness of the YMCA Virtuagym website from months 2 to 6 (Supplemental File 3 in Multimedia Appendix 3).

## Discussion

### Overview of Findings

Experiences with technology uptake and usage among adults aging with HIV were characterized by 4 components: (1) preparations for technology, (2) interactions with technology, (3) facilitators and satisfaction with technology, and (4) challenges and frustrations with technology. Experiences with technology interacted with intrinsic contextual factors (prior exposure linked to anticipated comfort or challenges with technology) and extrinsic contextual factors (COVID-19 pandemic; and technological and social support). Some participants experienced increasing technology burden and fatigue over time, others maintained neutral perspectives throughout, while others developed intentions for continued use postintervention.

### Experiences With Technology Uptake and Usage

Technology in the online CBE intervention included a combination of digital tools and platforms, including WPAMs (Fitbit Inspire 2), a videoconferencing platform (Zoom), webcams, the Sweat for Good YMCA app, and YMCA Virtuagym website. Participants demonstrated variation in experiences with technology uptake and usage across different technologies, with preferences emerging for specific types of technology and modes of delivery. Synchronous coaching sessions via Zoom achieved relatively high amounts of uptake following initial setup, likely facilitated by prior exposure gained during the COVID-19 pandemic [53]. The widespread adoption of Zoom during the COVID-19 pandemic likely created a foundational level of comfort that translated well to the synchronous coaching sessions, with participants expressing appreciation for the flexibility and immediate feedback available through this platform [26,53]. In contrast, participants in this study had variable experiences with the Sweat for Good YMCA app and WPAMs. While WPAMs were primarily used for real-time monitoring of physical activity metrics such as step counts and sleep patterns, the Sweat for Good YMCA app was used to administer asynchronous exercise content. Participants had varied preferences for synchronous or asynchronous tools that had implications for engagement with technology within the intervention. This has direct implications for considerations of technology uptake and usage in digital intervention design, suggesting the need to align online CBE interventions with user preferences for both the type of technology and the mode of delivery. Hence, it is important to recognize that adults aging with HIV may respond differently to synchronous tools that offer real-time human connection versus asynchronous tools that require greater independence [17,18,26].

All participants recruited for the telecoaching CBE study were required to have access to and comfort with the usage of technology. Despite all participants reporting comfort with different forms of technology at baseline, participants expressed challenges with uptake and usage of technology in the intervention. Of the 11 participants, 6 (66%) had prior exposure to a WPAM; however, this was insufficient for sustained uptake and usage of the Fitbit Inspire 2. Additionally, participants voiced inconsistent usage of more complex platforms, such as the Sweat for Good YMCA app. The disconnect between high baseline comfort and subsequent struggles with technology may reflect the requirement for participants to simultaneously navigate multiple technology platforms in the intervention that exceeded individual comfort with each component of the technology [18,26]. These findings suggest that technological complexity, rather than basic digital technology skills, posed a greater barrier to sustained use among adults living with HIV [39]. Future interventions should move beyond one-time onboarding to include tailored, continuous support that addresses and simplifies multiplatform navigation and adapts to participants’ evolving needs among adults aging with HIV [17,39,54]. Additionally, future CBE interventions may consider offering a broader range of WPAMs, such as commercially available smartwatches and fitness trackers. Tailoring digital health wearables based on individual preferences, digital health literacy, and accessibility may provide customizable feedback and enhance usability and sustained use over time.

Participants highlighted several facilitators and points of satisfaction with the technology used in the online CBE intervention, including flexible access to workouts, real-time interaction with personal trainers, and digital tools for self-monitoring physical activity. Zoom was consistently highlighted for its convenience and flexibility, supporting real-time interaction with personal trainers, accountability, and routine building, factors shown to improve exercise adherence among adults aging with HIV [17,54,55]. The WPAM served as a motivator for some, enabling self-monitoring of physical activity, steps, and sleep, which some participants found satisfying and empowering. Similar to prior studies, gamification features of WPAMs and real-time feedback supported users’ sense of progress and enhanced intrinsic motivation among adults aging with HIV [30,32,40]. The Sweat for Good YMCA app provided asynchronous, on-demand workouts that aligned with participants’ preferences for flexibility and autonomy, reinforcing the importance of choice between synchronous and asynchronous formats among adults aging with HIV [55]. These findings suggest that digital tools and processes can support engagement in exercise among adults aging with HIV when designed to be adaptable, user-friendly, and reinforced through personalized feedback and social accountability [18,41,55]. Future interventions should leverage these features by integrating reliable self-monitoring technologies and offering multiple modes of delivery to enhance engagement in online CBE interventions among adults aging with HIV.

Challenges and frustrations with technology use in this study included device syncing failures and navigation difficulties with the Sweat for Good YMCA app and connectivity problems with Zoom videoconferencing. WPAMs were valued for their feedback features but also generated frustration when step counts were perceived as inaccurate or syncing required repeated effort. This aligns with systematic measurement errors in consumer WPAM devices and the importance of balancing the motivational benefits of self-monitoring technology against potential user frustration from technical limitations for adults aging with HIV [40,56]. Participants described difficulty finding exercises to match their activity levels due to navigation difficulties on the Sweat for Good YMCA app. While Zoom was generally reliable, several participants reported audio lag and video freezing, issues commonly experienced in online digital health interventions with synchronous videoconferencing among adults aging with HIV [26,39,55]. Difficulties with device accuracy, navigation of fitness apps, and technical troubleshooting are widely reported in studies involving adults living with HIV using WPAMs or engaging in online exercise interventions [17,44,55]. While these technical experiences are not unique to this sample population, they occur within a context shaped by the challenges of aging with HIV, including health unpredictability, stigma, and the risk of loneliness [57-60]. Embedding lived experiences into intervention design and ongoing technical support are essential strategies to overcome digital barriers and promote uptake and usage with online CBE interventions among adults aging with HIV.

### Changes in Technology Experiences Over Time

Changes in experiences with technology over time demonstrated initial attitudes toward technology that did not always predict sustained use with the technology. Some participants experienced increasing technology burden and fatigue, others maintained neutral perspectives throughout, while others developed optimistic intentions for continued use postintervention. Participants used Zoom consistently throughout, as evidenced by consistent usability responses on the Telehealth Indicator Questionnaire, likely due to its relative simplicity, prior exposure, and use as a communication platform with their personal trainer. While the use of WPAMs and the Sweat for Good YMCA app may have declined, this may be attributed to persistent technical issues and limited adaptability. This mirrored patterns of engagement with digital health technology seen in adults aging with HIV [32,55,61]. Some participants reported feeling overwhelmed by syncing requirements, app navigation, and inconsistent device feedback in the online CBE intervention, factors that diminished motivation and created friction in maintaining regular technology use. This aligns with research that prolonged exposure to poorly optimized tools can lead to emotional and cognitive fatigue, especially among users managing multimorbidity [19,41]. Positive changes in technology use were most apparent in self-directed components, suggesting that asynchronous, exploratory technology use may become more rewarding over time as users gain confidence. For adults aging with HIV, these findings reinforce the need for sustained technical support and simplified interface design, as digital interventions must be responsive, flexible, and grounded in lived experience to facilitate long-term engagement in online CBE interventions [17,18,55].

### Strengths, Limitations, and Implications for Future Research

A strength of this study was the longitudinal observational design involving both interview and questionnaire data, allowing for an in-depth understanding of participants’ experiences with technology over time. Interview and questionnaire data were analyzed separately, with the questionnaires used descriptively to provide context to the interview findings. We conducted a collaborative content analysis among the team, allowing for diverse perspectives to be integrated throughout coding and interpretation. This iterative, team-based process supported reflexivity and rigor in the analysis. Additionally, input from community experts living with HIV helped ground the analysis in the lived experiences of the HIV community.

Our study sample size was fixed based on the existing data in the telecoaching CBE study. Reaching thematic saturation was not a goal of our study. However, in the results, we indicate the participants via participant number and time point alongside interview quotes to indicate the contribution of participant perspectives across the breadth of categories. Other strategies to enhance rigor included our inclusion of all available interview and questionnaire data for participants across the study. Collectively, we believe we were able to capture an ample description of participants’ perspectives related to experiences with uptake and usage, enabling us to achieve our study objectives.

Regarding study limitations, the interviews were not solely focused on technology; hence, the amount of data and depth of insights specific to technology use was limited. Not all participants completed interviews at T1 and T2 or the Telehealth Indicator Questionnaire at T2, which limited the ability to explore participants’ changes in technology experiences over time. Our sample was drawn from community-dwelling adults living with HIV in Toronto, a large urban center with strong digital infrastructure, limiting transferability to rural or remote settings with greater barriers to connectivity and access to the internet. Additionally, future work should examine sex and gender differences in technology experiences and engagement within online CBE interventions among adults aging with HIV to promote equitable access, engagement, and effectiveness [62].

### Conclusions

Experiences with technology uptake and usage among adults aging with HIV were characterized by 4 components, including preparations for technology use, interactions with the technology, facilitators and satisfaction with technology, and challenges and frustrations with technology. Experiences with technology interacted with intrinsic contextual factors (prior exposure linked to anticipated comfort or challenges with technology) and extrinsic contextual factors (COVID-19 pandemic and technological and social support). Among adults aging with HIV, changes in technology experiences over time highlighted the unpredictability of initial impressions, with some participants expressing technology fatigue and burden, whereas others maintained consistent attitudes of usage over time with intentions to continue using digital tools and processes beyond the intervention. Findings with experiences of technology uptake and usage in this study underscore the importance of incorporating personalized and tailored onboarding and sustained technological support with an online CBE intervention to promote engagement in physical activity among adults aging with HIV.

## Supplementary material

10.2196/86785 Multimedia Appendix 1 Interview guide (technology questions highlighted).

10.2196/86785 Multimedia Appendix 2 Telehealth indicator questionnaire.

10.2196/86785 Multimedia Appendix 3 Direction of change of participant responses to telehealth indicator questionnaire item responses at months 2 and 6.

10.2196/86785 Checklist 1 Standards for Reporting Qualitative Research checklist.

## Abbreviations

CBE: community-based exercise
WHO: World Health Organization
WPAM: wireless physical activity monitor

## References

[1] Canada’s progress towards ending the HIV epidemic, 2022. Public Health Agency of Canada.

[2] (2025). AIDS, crisis and the power to transform: UNAIDS global AIDS update 2025. https://www.unaids.org/sites/default/files/2025-07/2025-global-aids-update-JC3153_en.pdf.

[3] Hasse B, Ledergerber B, Furrer H (2011). Morbidity and aging in HIV-infected persons: the Swiss HIV cohort study. Clin Infect Dis.

[4] McArthur JC, Johnson TP (2020). Chronic inflammation mediates brain injury in HIV infection: relevance for cure strategies. Curr Opin Neurol.

[5] O’Brien KK, Bayoumi AM, Strike C, Young NL, Davis AM (2008). Exploring disability from the perspective of adults living with HIV/AIDS: development of a conceptual framework. Health Qual Life Outcomes.

[6] O’Brien KK, Hanna S, Gardner S (2014). Validation of the Episodic Disability Framework with adults living with HIV. Disabil Rehabil.

[7] Caspersen CJ, Powell KE, Christenson GM (1985). Physical activity, exercise, and physical fitness: definitions and distinctions for health-related research. Public Health Rep.

[8] Leach LL, Bassett SH, Smithdorf G, Andrews BS, Travill AL (2015). A systematic review of the effects of exercise interventions on body composition in HIV+ adults. Open AIDS J.

[9] Aminde JAA, Burton NW, Thng C, Clanchy K (2024). A systematic review and meta-analysis evaluating the effectiveness of minimally supervised home and community exercise interventions in improving physical activity, body adiposity and quality of life in adults living with HIV. Prev Med.

[10] O’Brien KK, Tynan AM, Nixon SA, Glazier RH (2017). Effectiveness of Progressive Resistive Exercise (PRE) in the context of HIV: systematic review and meta-analysis using the Cochrane Collaboration protocol. BMC Infect Dis.

[11] Gomes-Neto M, Saquetto MB, Alves IG, Martinez BP, Vieira JPB, Brites C (2021). Effects of exercise interventions on aerobic capacity and health-related quality of life in people living with HIV/AIDS: systematic review and network meta-analysis. Phys Ther.

[12] Bull FC, Al-Ansari SS, Biddle S (2020). World Health Organization 2020 guidelines on physical activity and sedentary behaviour. Br J Sports Med.

[13] Vancampfort D, Mugisha J, De Hert M (2018). Global physical activity levels among people living with HIV: a systematic review and meta-analysis. Disabil Rehabil.

[14] Vancampfort D, Mugisha J, De Hert M, Probst M, Stubbs B (2017). Sedentary behavior in people living with HIV: a systematic review and meta-analysis. J Phys Act Health.

[15] Simonik A, Vader K, Ellis D (2016). Are you ready? Exploring readiness to engage in exercise among people living with HIV and multimorbidity in Toronto, Canada: a qualitative study. BMJ Open.

[16] Johs NA, Kellar-Guenther Y, Jankowski CM, Neff H, Erlandson KM (2019). A qualitative focus group study of perceived barriers and benefits to exercise by self-described exercise status among older adults living with HIV. BMJ Open.

[17] Solomon P, Carusone SC, Davis AM, Aubry R, O’Brien KK (2021). Experiences of people living with HIV in community based exercise: a qualitative longitudinal study. J Int Assoc Provid AIDS Care.

[18] Lau B, Sharma I, Manku S (2022). Considerations for developing and implementing an online community-based exercise intervention with adults living with HIV: a qualitative study. BMJ Open.

[19] Montgomery CA, Henning KJ, Kantarzhi SR, Kideckel TB, Yang CFM, O’Brien KK (2017). Experiences participating in a community-based exercise programme from the perspective of people living with HIV: a qualitative study. BMJ Open.

[20] Li A, McCabe T, Silverstein E (2017). Community-based exercise in the context of HIV: factors to consider when developing and implementing community-based exercise programs for people living with HIV. J Int Assoc Provid AIDS Care.

[21] O’Brien KK, Davis AM, Chan Carusone S (2021). Examining the impact of a community-based exercise intervention on cardiorespiratory fitness, cardiovascular health, strength, flexibility and physical activity among adults living with HIV: a three-phased intervention study. PLoS ONE.

[22] Galea MD (2019). Telemedicine in rehabilitation. Phys Med Rehabil Clin N Am.

[23] Russell TG (2007). Physical rehabilitation using telemedicine. J Telemed Telecare.

[24] Peretti A, Amenta F, Tayebati SK, Nittari G, Mahdi SS (2017). Telerehabilitation: review of the state-of-the-art and areas of application. JMIR Rehabil Assist Technol.

[25] Jones J, Knox J, Meanley S (2022). Explorations of the role of digital technology in HIV-related implementation research: case comparisons of five ending the HIV epidemic supplement awards. J Acquir Immune Defic Syndr.

[26] Piraux E, Reychler G, Forget P, Yombi JC, Caty G (2019). Feasibility and preliminary effects of a telerehabilitation program for people living with HIV: a pilot randomized study. J Assoc Nurses AIDS Care.

[27] Loeckx M, Rabinovich RA, Demeyer H (2018). Smartphone-based physical activity telecoaching in chronic obstructive pulmonary disease: mixed-methods study on patient experiences and lessons for implementation. JMIR Mhealth Uhealth.

[28] Ramage ER, Fini NA, Lynch EA, Patterson A, Said CM, English C (2019). Supervised exercise delivered via telehealth in real time to manage chronic conditions in adults: a protocol for a scoping review to inform future research in stroke survivors. BMJ Open.

[29] Lai B, Bond K, Kim Y, Barstow B, Jovanov E, Bickel CS (2020). Exploring the uptake and implementation of tele-monitored home-exercise programmes in adults with Parkinson’s disease: a mixed-methods pilot study. J Telemed Telecare.

[30] Albergoni A, Hettinga FJ, La Torre A, Bonato M, Sartor F (2019). The role of technology in adherence to physical activity programs in patients with chronic diseases experiencing fatigue: a systematic review. Sports Med Open.

[31] Kapadia V, Ariani A, Li J, Ray PK (2015). Emerging ICT implementation issues in aged care. Int J Med Inform.

[32] Turner JR, Chow J, Cheng J (2023). Wireless physical activity monitor use among adults living with HIV in a community-based exercise intervention study: a quantitative, longitudinal, observational study. BMJ Open.

[33] Wright PJ, Raynor PA, Bowers D (2023). Leveraging digital technology for social connectedness among adults with chronic conditions: a systematic review. Digit Health.

[34] Berry ECJ, Sculthorpe NF, Warner A, Mather JD, Sanal-Hayes NEM, Hayes LD (2025). A scoping review of the feasibility, usability, and efficacy of digital interventions in older adults concerning physical activity and/or exercise. Front Aging.

[35] Ramage ER, Fini N, Lynch EA (2021). Look before you leap: interventions supervised via telehealth involving activities in weight-bearing or standing positions for people after stroke-a scoping review. Phys Ther.

[36] Nittas V, Zecca C, Kamm CP, Kuhle J, Chan A, von Wyl V (2023). Digital health for chronic disease management: an exploratory method to investigating technology adoption potential. PLoS ONE.

[37] Nittas V, Daniore P, Chavez SJ, Wray TB (2024). Challenges in implementing cultural adaptations of digital health interventions. Commun Med (Lond).

[38] Apolinário-Hagen J, Menzel M, Hennemann S, Salewski C (2018). Acceptance of mobile health apps for disease management among people with multiple sclerosis: web-based survey study. JMIR Form Res.

[39] Campbell BR, Ingersoll KS, Flickinger TE, Dillingham R (2019). Bridging the digital health divide: toward equitable global access to mobile health interventions for people living with HIV. Expert Rev Anti Infect Ther.

[40] Dagenais M, Cheng D, Salbach NM, Brooks D, O’Brien KK (2019). Wireless physical activity monitor use among adults living with HIV: a scoping review. Rehabil Oncol.

[41] Oursler KK, Marconi VC, Briggs BC, Sorkin JD, Ryan AS, FIT VET Project Team (2022). Telehealth exercise intervention in older adults with HIV: protocol of a multisite randomized trial. J Assoc Nurses AIDS Care.

[42] O’Brien KK, Ibáñez-Carrasco F, Carusone SC (2023). Piloting an online telecoaching community-based exercise intervention with adults living with HIV: protocol for a mixed-methods implementation science study. BMJ Open.

[43] Rourke SB, Gardner S, Burchell AN (2013). Cohort profile: the Ontario HIV Treatment Network Cohort Study (OCS). Int J Epidemiol.

[44] Ibáñez-Carrasco F, McDuff K, Da Silva G (2025). Qualitative insights from an online community-based exercise intervention for persons living with HIV. Front Rehabil Sci.

[45] Huggins AC, Ritzhaupt AD, Dawson KM (2014). Measuring information and communication technology literacy using a performance assessment: validation of the student tool for technology literacy (ST2L). Comput Educ.

[46] (2014). Manual for measuring ICT access and use by housholds and individuals. International Telecommunication Union.

[47] Molnar A, Weerakkody V (2013). IFIP International Federation for Information Processing.

[48] Flicker S, Nixon SA (2015). The DEPICT model for participatory qualitative health promotion research analysis piloted in Canada, Zambia and South Africa. Health Promot Int.

[49] (2018). NVivo qualitative data analysis software. Version 12. QSR International.

[50] (2016). Microsoft Excel. Microsoft Corporation.

[51] Flynn A, Preston E, Dennis S, Canning CG, Allen NE (2023). Utilising telehealth to support exercise and physical activity in people with Parkinson disease: a program evaluation using mixed methods. BMC Health Serv Res.

[52] Dunston ER, Malouf A, Podlog LW (2025). Experiences participating in a telehealth exercise program among older adults with cancer: a qualitative study. J Cancer Surviv.

[53] Budak JZ, Scott JD, Dhanireddy S, Wood BR (2021). The impact of COVID-19 on HIV care provided via telemedicine-past, present, and future. Curr HIV/AIDS Rep.

[54] Solomon P, Chan Carusone S, Davis AM, Aubry R, O’Brien KK (2021). A qualitative study of fitness coaches’ experiences in community based exercise with people living with HIV. J Int Assoc Provid AIDS Care.

[55] Jiancaro T, Bayoumi AM, Ibáñez-Carrasco F (2023). Factors influencing initial implementation of an online community-based exercise intervention with adults living with HIV: a systems approach. Front Rehabil Sci.

[56] Dagenais M, Salbach NM, Brooks D, O’Brien KK (2020). Assessing the measurement properties of the Fitbit Zip® among adults living with HIV. J Phys Act Health.

[57] Solomon P, O’Brien K, Wilkins S, Gervais N (2014). Aging with HIV and disability: the role of uncertainty. AIDS Care.

[58] Yoo-Jeong M, Hepburn K, Holstad M, Haardörfer R, Waldrop-Valverde D (2020). Correlates of loneliness in older persons living with HIV. AIDS Care.

[59] Grov C, Golub SA, Parsons JT, Brennan M, Karpiak SE (2010). Loneliness and HIV-related stigma explain depression among older HIV-positive adults. AIDS Care.

[60] Pollak C, Cotton K, Winter J, Blumen H (2025). Health outcomes associated with loneliness and social isolation in older adults living with HIV: a systematic review. AIDS Behav.

[61] Ibáñez-Carrasco F, Jiancaro T, Torres B (2023). HIV in MOTION: a community of practice on physical rehabilitation for and by people living with HIV and their allies. Front Rehabil Sci.

[62] Sahel-Gozin N, Loutfy M, O’Brien KK (2023). Exploring experiences engaging in exercise from the perspectives of women living with HIV: a qualitative study. PLoS One.

