# Supplementary material for: Experiences With Technology Among Adults Aging With HIV Engaged in an Online Community–Based Exercise Intervention Study: Longitudinal Qualitative Descriptive Study and Secondary Data Analysis
Source: JMIR Rehabil Assist Technol. 2026 Jul 2;13:e86785. doi: 10.2196/86785 (PMC13327373; doi:10.2196/86785)
Supplement: Multimedia Appendix 1 [file rehab-v13-e86785-s001.pdf]

Supplemental File 1. Interview guide with highlighted questions relating to technology

INTERVIEW GUIDE with Adults living with HIV

Thank you for agreeing to participate in this study. I am meeting with you over the course of this study, to try to get a better understanding of your experience within the Tele-Coaching CBE Intervention, specifically your thoughts on any changes in your health *outcomes* (objective 2) as well as the *process (Implementation & Adoption)* (objective 4) of engaging in Tele-Coaching CBE. This part of the study involves participating in 3 interviews over the course of the Tele-Coaching CBE Intervention Study.

Overview of Interviews

Initiation of the CBE Intervention Interview: In this first interview at baseline, just before you start the CBE intervention, we would like to get a sense of a) your *experience* with exercise to date, b) any *goals or anticipated benefits* of participating in the upcoming CBE intervention, and c) any *concerns or perceived challenges* that you might have leading up to the start of the CBE intervention.

After CBE Intervention Interview: The second interview will occur when after you complete the exercise intervention. In this interview, we would like to get a better understanding of a) the *process* of participating in the Tele-coaching CBE intervention (objective 4), b) any *perceived changes* (either benefits or harms) of the Tele-Coaching CBE intervention on your health and disability and your engagement in the care cascade since you started the exercise program (objective 2), c) strengths and challenges taking part in the Tele coaching CBE intervention (objective 4), and c) your intention to continue to *engage* in the CBE post-intervention (objective 3). We are specifically interested in your experiences, strengths, challenges of use of the technology for the intervention.

After the Follow-Up Maintenance Phase Interview (End of Study): The third and final interview will occur after you complete the entire study. In this interview, we would like to obtain your thoughts on the overall a) *process* (strengths and challenges) of participating in the CBE study (objective 4), b) any further updates on the *perceived impact* (benefits or harms) of Tele-Coaching CBE on your health and engagement in the care cascade over time (objective 2), and c) your *intention* to continue to engage in CBE post study (objective 3). Again, we are interested in your experiences, strengths, challenges of use of the technology for the intervention. In addition, in the second and third interviews, we will specifically explore the *influence of extrinsic factors* such as social support or stigma and *intrinsic factors* such as concurrent health conditions, gender, age and coping strategies [contextual factors in the *Episodic Disability Framework]* on the impact and your level of engagement or adherence with exercise. We will also ask you about your experience and ability of using the intervention online (tele-coaching) and the technology. We are interested in learning about what you think makes it easier or harder to access and participate in tele-coaching community based exercise and whether there should be any refinements made to this Tele-Coaching CBE intervention before more broadly implementing with the community. Results from these interviews will be used for the future refinement of an accessible, feasible, safe, and sustainable Tele-Coaching CBE program for people living with HIV. Before we start, do you have any questions?

INTERVIEW #1 - BASELINE (INITIATION OF CBE) INQURY – People Living with HIV Participants

1. Are you currently ***living with any health related challenges with HIV?*** If so, can you describe what they are? (physical, social, mental, cognitive, uncertainty).

2. What is your ***current experience with exercise***?
    a. Are you currently engaging in regular exercise?
    b. If so, can you describe the type, frequency, duration, intensity?

3. Do you have any ***goals*** for participating in the CBE intervention?
    a. If so, what are they?
        i. Physical health goals? Mental health goals?
    b. How do you think you might best achieve them?

4. Do you ***anticipate any benefits*** from participating in the CBE Intervention?
    a. If so what might they be?
        i. Improvements in health or reduction of disability? (mental health, physical health, social inclusion, uncertainty, cognitive health)
        ii. Increased social interaction with others
        iii. Reduced feelings of stigma (specifically with ability to exercise at home).

5. Do you have any ***concerns or perceived challenges*** of participating in the upcoming CBE intervention?
    a. Inability to use the tele-coaching technology
    b. Concerns with exercising at home
    c. Inability to keep up with the exercise
    d. Fitbit
    e. Inability to attend regularly
    f. Feelings of stigma
    g. Potential for episodes of illness (or other life triggers that may arise)
    h. Long term feasibility of engaging in an exercise program over time
    i. Concerns with technology
    j. Concerns with privacy of exercising in the home

6. How do you ***feel about adopting the technology*** necessary for the upcoming online CBE tele-coaching intervention?
    a. Concerns with using the technology (use, confidentiality, connection etc.)
    b. Comfort level using the technology
    c. Willingness to learn to use the technology
    d. Confidence in using the technology

INTERVIEW #2 – POST CBE INTERVENTION LINE OF INQUIRY – People Living with HIV

Perceived Impact of Exercise from perspective of participants [objective 2]

1. Can you describe your ***experience*** so far participating in the Tele-Coaching CBE intervention?
    a. How did you come to access **the study** (or start up)?
    b. Why did you sign up for the study? What was appealing about it?
        i. **CBE Intervention – One-on-one Coaching**
            - Can you tell me what it is like to be involved in the one-on-one coaching as part of the of the CBE intervention?
        ii. ***Technology – Tele-coaching - CBE Intervention***
            - Can you tell me what it was like being coached by your fitness instructor online?
        iii. **CBE Intervention – Exercise Program**
            - Can you tell me what it was like engaging in exercise at your house?
            - Have you accessed the online group classes via Sweat for Good App?
            - Have you been exercising at the YMCA (if open)? Or exclusively at home?
        iv. **Self-Management Sessions (online or in person)**
            - Did you attend the self-management sessions online or in person?

- Can you tell me what it is like to be involved in the self-management sessions with the CBE intervention?

v. **Fitbit Inspire 2**

- Did you regularly wear your Fitbit Inspire 2?
- Did you use your Fibit Inspire 2 as a way to track your physical activity?
- Did you find wearing your Fitbit Inspire 2 motivated you to exercise?

c. What has it been accessing the **fitness coaching sessions online at home**?
d. What ***types of activities*** are you engaging in?
e. Does the ***individual nature of the coaching sessions*** make a difference (one-on-one engagement)? If so, how?
f. What has your ***attendance*** (or adherence) been like so far with the CBE intervention (online tele-coaching sessions, 3x weekly exercise, self-management sessions, Fitbit Inspire 2)? Are you attending your coaching sessions/YMCA/Exercising at home regularly? If not, why not? If yes, what are some of the reasons you're attending regularly?

Fidelity (adherence; dose or amount of intervention delivered; and quality of delivery (e.g. tele-coaching).

2. Can you walk through and describe **<u>your most recent coaching session</u>** from <u>start to finish</u>?
3. Can you walk through and explain the events of **your most recent independent exercise session** from <u>start to finish</u>?

Group versus Individualized Sessions

g. Were you engaged in any online 'group-based activities' for your exercise program apart from your coaching sessions?
h. Have you met / interacted with any other CBE participants? If so, can you describe? (For instance, are you running into other CBE participants at the YMCA (if applicable)?
i. Did you know them before the study, or did you meet them as part of your involvement in the study?).
j. Can you describe your exchanges (if any) with the other participants in the study?
k. Overall, can you comment on the extent to which you feel there is potentially a 'sense of community' developed (or not) with your peer and other CBE participants? If so, how? What has changed?

4. Have you noticed ***any changes in your health or disability (e.g. benefits or harms)*** since starting the intervention? If so, what were they? Overall, what do you think the ***impact of the tele-coaching CBE intervention*** has been for you thus far? [objective #2]

   Probe: When we met last time, you mentioned [*REFER TO BASELINE TRANSCRIPT*], has anything changed since then? If so, how has it changed?

   a. Physical health
   b. Mental health
   c. Cognitive health
   d. Social inclusion
   e. Daily functional activities
   f. Uncertainty or worrying about the future

5. Have there been any ***environmental factors*** that have influenced your experience with the tele-coaching CBE intervention? If so, can you describe? How might they influence the impact of exercise on your health?

   a. Social support (support from friends and family to engage in exercise; support from health providers; fitness instructors)

b. Stigma (reduction of stigma engaging in tele-coaching or exercising in group with other PHAs)
c. Feasibility of technology using online tele-coaching
d. Interactions with coach (Are they progressing the exercises? Are they correcting your form during the exercises? Did you feel motivated to see your coach each session?)
e. Interactions with research staff
f. Wearing a Fitbit

6. Have there been any ***personal factors*** that have influenced your experience with the tele-coaching CBE intervention? (either your ability to participate in the exercise; or the changes you might see with your health)? If so, can you describe? How might they influence the impact of exercise on your health?
   a. Personal attributes (age, gender, ethnocultural background, length of time since diagnosis)
      i. How has your ***gender*** influenced your ability to engage in exercise?
   b. Influence of other concurrent health conditions
   c. Ability to cope with HIV, living strategies (nutrition, medication adherence, smoking, etc)

Tele-Coaching Technology Experiences

7. How would you ***describe your experience using the technology*** as part of the CBE Tele-coaching intervention?
   a. Usability and ease of usage of technology
      i. Confidence in use
      ii. Comfort level
      iii. Ease of navigation
      iv. Learnability of the technology over time
   b. Satisfaction with technology
      i. Quality (visual, sound etc.)
      ii. Effectiveness of delivering coaching sessions
      iii. Engagement with coach through technology
      iv. Ability to understand coaches instructions through the technology
   c. Reliability of the technology
      i. Online technology connection
      ii. Confidence in technology to deliver your coaching session
      iii. Interruptions
      iv. Responsiveness of technology (was there any technological delays or pauses?)
      v. Confidentiality or privacy concerns
   d. Feasibility
      i. Would you continue to use technology for fitness coaching in the future? Why or why not?

8. Did you ***access the online YMCA group classes via the Sweat for Good App***? If yes, how was your experience with the class through this technology?
   a. Usability and ease of usage of online classes
   b. Satisfaction with the online classes (visual and sound quality)
   c. Reliability of the technology (connection, interruptions, responsiveness)
   d. Confidentiality of the online classes
   e. Feasibility of the online classes
   f. Future online class use

Process of CBE Translation – Including Adoption and Implementation [objective 4]
Questions adapted for RE-AIM Planning Tool - series of 'thought questions' which serve as a checklist for key issues to consider when evaluating the intervention.

9. Have there been any ***strengths or challenges*** associated with the translation of the tele-coaching CBE intervention into the HIV community [objective 4]? (e.g. What aspects of the CBE intervention do you like? Not like?)
    a. Online tele-coaching technology (feasibility, use, comfort, satisfaction)
    b. Online fitness instructors
    c. Timing of the exercise sessions
    d. Online exercise classes
    e. Accessibility of exercising on your own at home
    f. Monthly self-management education sessions
    g. Equipment (Fitbit)
    h. Engaging with other participants
    i. Stigma
    j. Ability to keep up with the sessions
    k. Ability to attend the sessions regularly
    l. Experiences of episodic illness as a trigger that might influence ability to exercise

10. What are your thoughts on your ***ability and willingness to continue to engage in exercise over time?***
    a. Do you plan to continue with the exercise in the next phase? If yes, why? If not, why not?
    b. ***When we met last time, you mentioned [REFER TO BASELINE TRANSCRIPT], has anything changed since then?* If so, how has it changed?**

11. How confident are you that you will be ***able to adopt exercise*** into part of your lifestyle over the long term? Can you describe in more detail?
    a. ***When we met last time, you mentioned* [*REFER TO BASELINE TRANSCRIPT*], *has anything changed since then?* If so, how has it changed?**

12. How confident are you that the intervention (or program) can be ***consistently delivered*** (or implemented) as it is intended?
    a. What might be the greatest threat **(or challenge)** to consistently implementing a tele-coaching CBE program in the community? Do you have any ideas on how to overcome these barriers?

13. How confident are you that other community-based organizations (such as other YMCAs or other community organizations) may be ***willing and able to offer*** this tele-coaching intervention as a fully developed program?
    a. What do you think will be the greatest barriers to other sites or organizations adopting this tele-coaching intervention? Do you have any ideas on how to overcome these barriers?1

14. Do you have any suggestions for ways in which to ***sustain the tele-coaching CBE program*** over the long term in the HIV community?

15. Do you have suggestions on ***other partnerships or stakeholders*** that should be considered in the long term sustainability of a tele-coaching CBE program? If so, who are they?
