# Supplementary material for: Experiences With Technology Among Adults Aging With HIV Engaged in an Online Community–Based Exercise Intervention Study: Longitudinal Qualitative Descriptive Study and Secondary Data Analysis
Source: JMIR Rehabil Assist Technol. 2026 Jul 2;13:e86785. doi: 10.2196/86785 (PMC13327373; doi:10.2196/86785)
Supplement: Multimedia Appendix 2 [file rehab-v13-e86785-s002.pdf]

Supplemental File 2 – Telehealth Indicator Questionnaire

Usability / Ease of Use				
**Statement**	**Check the box that describes the usability**			
1) The online tele-coaching system was **easy to use** overall	Not easy to use at all (very hard) ☐	Somewhat easy to use (a little hard) ☐	Easy to use ☐	Very easy to use ☐
2) The online tele-coaching system was **easy to navigate**	Not easy to navigate at all ☐	Somewhat easy to navigate ☐	Easy to navigate ☐	Very easy to navigate ☐
3) The **help features were helpful** when needed	Not helpful at all ☐	Somewhat helpful ☐	Helpful ☐	Very helpful ☐
4) The online tele-coaching system was **simple to understand**	Not simple at all (very complicated) ☐	Somewhat simple (a little complicated) ☐	Simple ☐	Very simple ☐
**Comments:**				

Satisfaction with Telehealth – the Online CBE Intervention (online technology)*				
How satisfied were you with:	**Check the box that describes your satisfaction**			
5) The **voice quality** of the equipment.	Excellent (4) ☐	Good (3) ☐	Fair (2) ☐	Poor (1) ☐
6) The **visual quality** of the equipment.	Excellent (4) ☐	Good (3) ☐	Fair (2) ☐	Poor (1) ☐
7) Your **personal comfort** in using the online CBE system.	Excellent (4) ☐	Good (3) ☐	Fair (2) ☐	Poor (1) ☐
8) The **ease of connecting** with the fitness instructor	Excellent (4) ☐	Good (3) ☐	Fair (2) ☐	Poor (1) ☐
9) The **length of time** with the online CBE system / fitness instructor	Excellent (4) ☐	Good (3) ☐	Fair (2) ☐	Poor (1) ☐

10) The **explanation of your program** by the fitness instructor	Excellent (4) ☐	Good (3) ☐	Fair (2) ☐	Poor (1) ☐
11) The **thoroughness, carefulness and skillfulness** of the fitness instructor	Excellent (4) ☐	Good (3) ☐	Fair (2) ☐	Poor (1) ☐
12) The **courtesy, respect, sensitivity, and friendliness** of the fitness instructor and research team	Excellent (4) ☐	Good (3) ☐	Fair (2) ☐	Poor (1) ☐
13) How well your **privacy was respected**	Excellent (4) ☐	Good (3) ☐	Fair (2) ☐	Poor (1) ☐
14) How well the research team **answered your questions about the equipment**	Excellent (4) ☐	Good (3) ☐	Fair (2) ☐	Poor (1) ☐
**Comments:**				

**Reliability of tele-health (online technology)**				
**Statement**	**Check the box that describes the reliability of the online technology**			
15) There were **interruptions** while using the online coaching technology	Many interruptions ☐	A few interruptions ☐	One or two interruptions ☐	No interruptions ☐
16) I had to **reboot** the online tele-coaching system while using it	Had to reboot it many times ☐	Had to reboot it a few times ☐	Had to reboot it one or two times ☐	Never had to reboot it ☐
17) The online tele-coaching system was **responsive to my commands** (there were no delays, responded to changing settings etc.)	Not responsive ☐	A little responsive ☐	Responsive ☐	Very responsive ☐
**Comments:**				

TeSS Scale: *Score range: 10-40 (higher scores indicate greater satisfaction).

References:

Telehealth Satisfaction Scale (TeSS) (10 items): Items 5-14 adapted from: Morgan DG, Kosteniuk J, Stewart N, O'Connell ME, Karunanyake C, Beever R. The Telehealth Satisfaction Scale (TeSS): Reliability, validity, and satisfaction with telehealth in a rural memory clinic population. *Telemed J E Health.* 2014 Nov 1;20(11):997-1003.

Molnar A, Weerakkody V. Defining key performance indicators for evaluating the use of high definition video-to-video-services in eHealth. *IFIP International Federation for Information Processing*. 2013;AICT 412:452-461.
