# Supplementary material for: Experiences With Technology Among Adults Aging With HIV Engaged in an Online Community–Based Exercise Intervention Study: Longitudinal Qualitative Descriptive Study and Secondary Data Analysis
Source: JMIR Rehabil Assist Technol. 2026 Jul 2;13:e86785. doi: 10.2196/86785 (PMC13327373; doi:10.2196/86785)
Supplement: Multimedia Appendix 3 [file rehab-v13-e86785-s003.docx]

**Supplemental File 3.** Direction of change of participant responses to the Telehealth Indicator Questionnaire item responses at month 2 and 6 (n=8).

| **Criterion** | **Participant (n=8)** | | | | | | | | **Positive direction of change in item response**  **N (%)** | **Negative direction of change in item response**  **N (%)** | **No change in item response**  **N (%)** |
| --- | --- | --- | --- | --- | --- | --- | --- | --- | --- | --- | --- |
|  | P1 | P2 | P3 | P4 | P7 | P8 | P10 | P11 |  |  |  |
| **Usability (7 items)** | | | | | | | | | | | |
| Fitbit | – | – | + | Ø | – | Ø | – | Ø | 1 (13) | 4 (50) | 3 (38) |
| YMCA Virtuagym Website | – | Ø | Ø | Ø | – | Ø | Ø | + | 1 (13) | 2 (25) | 5 (63) |
| Sweat for Good App | Ø | Ø | – | Ø | Ø | + | Ø | + | 2 (25) | 1 (13) | 5 (63) |
| Personal training via Zoom | Ø | Ø | Ø | Ø | Ø | Ø | – | Ø | -- | 1 (13) | 7 (88) |
| Group online YMCA classes via Zoom | Ø | Ø | Ø | Ø | Ø | Ø | Ø | + | 1 (13) | -- | 7 (88) |
| Self-management sessions via Zoom | Ø | Ø | Ø | – | – | Ø | Ø | Ø | -- | 2 (25) | 6 (75) |
| Self-directed learning modules | Ø | + | Ø | + | + | + | – | + | 5 (63) | 1 (13) | 2 (25) |
| **Helpfulness (6 items)** | | | | | | | | | | | |
| Fitbit | + | – | – | Ø | – | Ø | Ø | + | 2 (25) | 3 (38) | 3 (38) |
| YMCA Virtuagym Website | – | – | Ø | – | – | Ø | Ø | + | 1 (13) | 4 (50) | 3 (38) |
| Sweat for Good App | Ø | – | – | – | Ø | + | Ø | + | 2 (25) | 3 (38) | 3 (38) |
| Zoom | + | – | Ø | Ø | Ø | Ø | Ø | Ø | 1 (13) | 1 (13) | 6 (75) |
| Self-management sessions via Zoom | + | Ø | + | – | – | + | Ø | Ø | 3 (38) | 2 (25) | 3 (38) |
| Self-directed learning modules | Ø | Ø | + | + | + | + | – | + | 5 (63) | 1 (13) | 2 (25) |
| **Satisfaction (6 items)** | | | | | | | | | | | |
| Voice quality of sessions | Ø | – | Ø | Ø | + | Ø | – | + | 2 (25) | 2 (25) | 4 (50) |
| Visual quality of sessions | Ø | – | Ø | Ø | + | Ø | – | + | 2 (25) | 2 (25) | 4 (50) |
| Personal comfort in using online CBE technology | Ø | – | Ø | Ø | + | Ø | – | + | 2 (25) | 2 (25) | 4 (50) |
| Ease of connecting with fitness instructor | + | Ø | – | Ø | + | Ø | Ø | + | 3 (38) | 1 (13) | 4 (50) |
| How well privacy was respected | – | Ø | – | Ø | + | Ø | Ø | Ø | 1 (13) | 2 (25) | 5 (63) |
| How well research team answered questions about equipment and technology | – | – | Ø | Ø | + | Ø | Ø | Ø | 1 + (13) | 2 (25) | 5 (63) |
| **Reliability (3 items)** | | | | | | | | | | | |
| Frequency of interruptions while using technology | Ø | – | Ø | Ø | + | Ø | Ø | Ø | 1 (13) | 1 (13) | 6 (75) |
| Frequency of reboots while using technology | Ø | Ø | Ø | Ø | Ø | Ø | + | – | 1 (13) | 1 (13) | 6 (75) |
| Technology responsiveness to commands | + | Ø | – | – | + | Ø | – | Ø | 2 (25) | 3 (38) | 3 (38) |
| **Direction of Change** | **Participant (n=8)** | | | | | | | |  |  |  |
|  | P1 | P2 | P3 | P4 | P7 | P8 | P10 | P11 |  |  |  |
| **Positive direction of change across all 22 items**  **Number of items (%)** | 5 (23) | 1  (5) | 3 (14) | 2  (9) | 10 (46) | 5 (23) | 1  (5) | 12 (55) |  |  |  |
| **Negative direction of change across all 22 items**  **Number of items (%)** | 5 (23) | 10 (46) | 6 (27) | 5 (23) | 6 (27) |  | 8 (36) | 1  (5) |  |  |  |
| **No change across all 22 items**  **Number of items (%)** | 12 (55) | 11 (50) | 13 (59) | 15 (68) | 6 (27) | 17 (77) | 13 (59) | 9  (41) |  |  |  |

+: Positive direction of change; -: Negative direction of change; Ø: No change.

Note: 3 participants did not complete both timepoints, therefore change in responses could not be analyzed.
